# Demographic profiles and environmental drivers of variation relate to individual breeding state in a long-lived trans-oceanic migratory seabird, the Manx shearwater

**DOI:** 10.1371/journal.pone.0260812

**Published:** 2021-12-16

**Authors:** Matt J. Wood, Coline Canonne, Aurélien Besnard, Shelly Lachish, Stace M. Fairhurst, Miriam Liedvogel, Dave Boyle, Samantha C. Patrick, Simon Josey, Holly Kirk, Ben Dean, Tim Guilford, Robin M. McCleery, Chris M. Perrins, Cat Horswill

**Affiliations:** 1 School of Natural & Social Sciences, University of Gloucestershire, Cheltenham, United Kingdom; 2 CEFE, Univ Montpellier, CNRS, EPHE-PSL University, IRD, Univ Paul Valéry Montpellier 3, Montpellier, France; 3 Edward Grey Institute, Department of Zoology, University of Oxford, Oxford, United Kingdom; 4 National Oceanography Centre, University of Southampton, Southampton, United Kingdom; 5 Oxford Navigation Group, Department of Zoology, University of Oxford, Oxford, United Kingdom; 6 ZSL Institute of Zoology, London, United Kingdom; 7 Centre for Biodiversity and Environmental Research, Department of Genetics, Evolution and Environment, University College London, London, United Kingdom; 8 Department of Zoology, University of Cambridge, Cambridge, United Kingdom; MARE – Marine and Environmental Sciences Centre, PORTUGAL

## Abstract

Understanding the points in a species breeding cycle when they are most vulnerable to environmental fluctuations is key to understanding interannual demography and guiding effective conservation and management. Seabirds represent one of the most threatened groups of birds in the world, and climate change and severe weather is a prominent and increasing threat to this group. We used a multi-state capture-recapture model to examine how the demographic rates of a long-lived trans-oceanic migrant seabird, the Manx shearwater *Puffinus puffinus*, are influenced by environmental conditions experienced at different stages of the annual breeding cycle and whether these relationships vary with an individual’s breeding state in the previous year (i.e., successful breeder, failed breeder and non-breeder). Our results imply that populations of Manx shearwaters are comprised of individuals with different demographic profiles, whereby more successful reproduction is associated with higher rates of survival and breeding propensity. However, we found that all birds experienced the same negative relationship between rates of survival and wind force during the breeding season, indicating a cost of reproduction (or central place constraint for non-breeders) during years with severe weather conditions. We also found that environmental effects differentially influence the breeding propensity of individuals in different breeding states. This suggests individual spatio-temporal variation in habitat use during the annual cycle, such that climate change could alter the frequency that individuals with different demographic profiles breed thereby driving a complex and less predictable population response. More broadly, our study highlights the importance of considering individual-level factors when examining population demography and predicting how species may respond to climate change.

## Introduction

As global climate change continues [1, 2], reliably predicting how species and populations are likely to respond becomes a key conservation priority. In wide-ranging and migratory species, such as seabirds, vulnerability to climate change may increase at different points during the breeding cycle. Seabirds as a group are also highly threatened by climate change, as well as severe weather events [3, 4]. Consequently, understanding how climate variables in the breeding and non-breeding foraging areas influence the demography of these species represents an important step in predicting how they are likely to respond.

Identifying environmental drivers of demography typically requires a strong understanding of a species habitat use. This is complicated in seabirds because they are unobservable throughout much of their annual cycle, most obviously during the non-breeding season. As a result, many studies investigating associations between environmental variation and seabird demography use large-scale climatic and oceanographic indices, such as the North Atlantic Oscillation (NAO) and El Niño Southern Oscillation (ENSO) (for example see references in [5, 6]). These large-scale climate indices can be advantageous when dealing with wide-ranging species because they capture variation in local weather conditions over large spatial scales [7]. However, individuals respond principally to local conditions, and therefore information on local variables is also typically needed to understand the direct and indirect mechanisms linking large-scale climate indices to an ecological response, such as demographic change [7].

The use of biologging devices to track individual movement in seabirds is now widespread, and the wide range of devices now available means that it is possible to track individuals throughout the year to ascertain habitat use during different phases of the annual cycle [8]. Studies using these data to extract local weather conditions at key phases of the annual cycle and relate them to demography are also now increasing (e.g. [9]). However, seabird movement studies have also shown that an individual’s breeding performance or status can affects its foraging range, for example failed and non-breeders are relieved of restricted central place constraints associated with chick provisioning and leave the colony earlier than successful breeders [10–12]. Individual differences in the timings of migration could therefore generate spatio-temporal variation in exposure to local weather conditions within a population, thereby bolstering or hindering subsets of individuals and altering interannual demography, as well as population composition [13–16].

**Fig 1:**
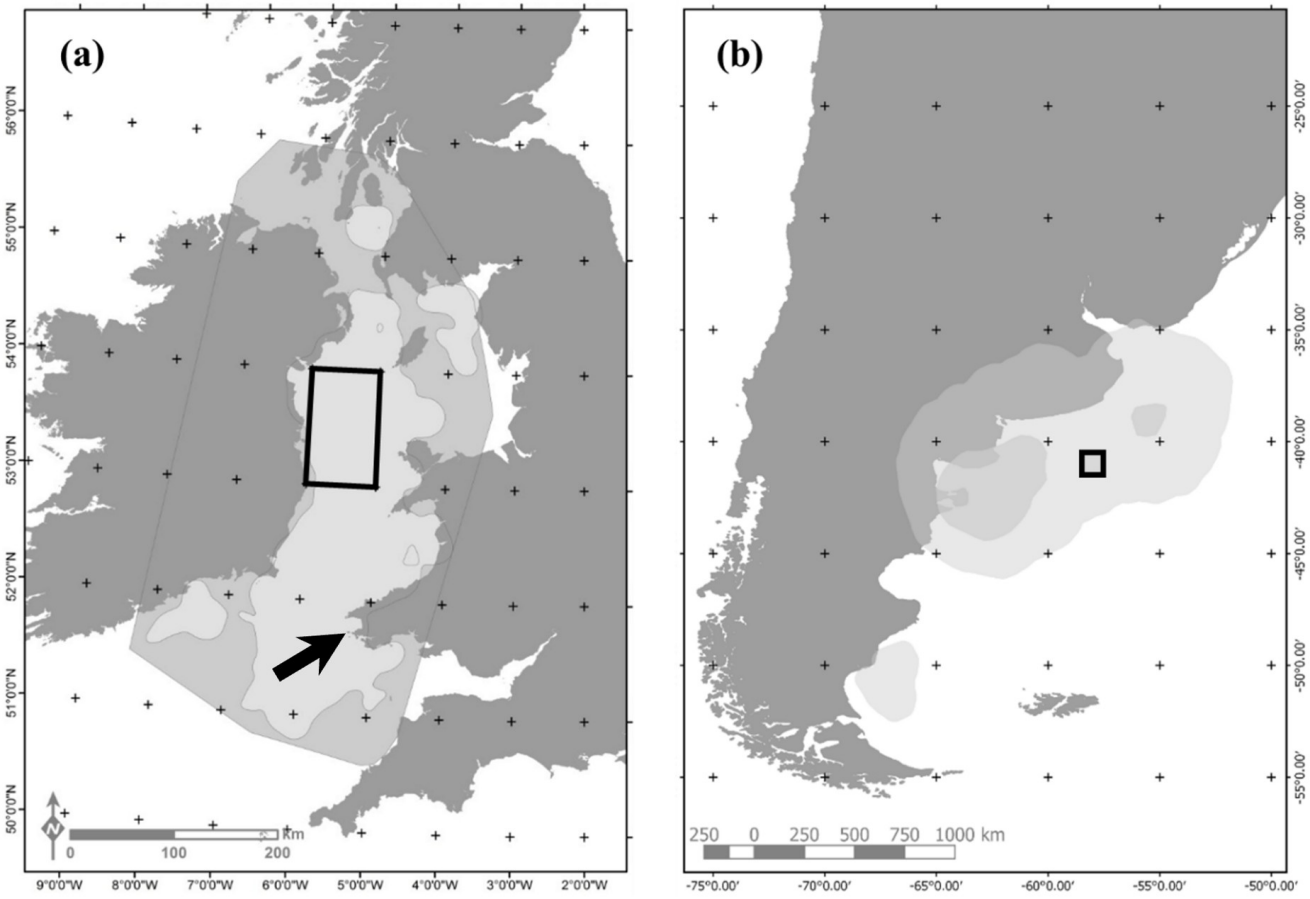
Key foraging areas used by Manx shearwaters breeding on Skomer Island, during the (a) breeding and (b) non-breeding season. Grey shading represents 90 and 95% kernel densities calculated from tracking of more than 100 individuals at an adjacent study plot on Skomer Island [17, 18], 50m from the Isthmus study plot. Black squares show the area used to extract localised environmental variables.

In this study, we examine whether individual differences in breeding state may alter spatio-temporal exposure to local weather conditions and generate heterogeneity in the demography of a trans-Atlantic migrant seabird, the Manx shearwater *Puffinus puffinus*. To do this, we use key areas of breeding and non-breeding habitat use for a population of Manx Shearwaters breeding on Skomer Island, Wales, UK, to extract local- and large-scale climate variables at relevant spatial and temporal scales. The year-round distribution of this population, including summer breeding season in the Irish Sea and wintering grounds off the coast of Argentina and Uruguay has been characterised using remote sensing technology (Fig 1; [17, 18]). We then used a multi-state capture-recapture model to identify climate drivers of survival, breeding propensity and breeding success, and test whether these relationships relate to an individual’s breeding status in the previous year (i.e., successful breeder, failed breeder and non-breeder).

## Methods

### Study species and site

Manx shearwaters are medium-sized (ca. 400g) long-lived pelagic seabirds with major colonies occurring on the Atlantic coast of the UK and Ireland [19]. Birds breed during the boreal summer, arriving at breeding colonies between late February and early March. Breeding pairs are socially monogamous, nesting in burrows excavated from the earth. Eggs are laid in early May and incubated for ca. 50 days, with chicks fledging ca. 70 days later between mid-August and late September [20]. Manx shearwaters are nocturnal at their breeding colonies, returning exclusively at night [20]. Outside of the breeding season, birds breeding at colonies in the UK share wintering grounds in the South-West Atlantic [18].

Skomer Island, Wales, UK, is estimated to support 350,000 breeding pairs of Manx shearwaters, representing approximately 42% and 39% of the UK and global breeding populations, respectively [21]. Demographic monitoring of Manx shearwaters at Skomer Island began in 1977 with a capture-recapture program to estimate apparent adult survival. Individual breeding status was then added to this program in 1993. Around 100 nest burrows are monitored annually in a study plot on The Isthmus (51.7368 N, 5.2808 W), a narrow strip of land (ca. 20m x 100m) that joins the two larger parts of Skomer island. Study burrows are marked and followed in subsequent years for as long as they are occupied, with 10–15 new study burrows added annually. The study plot was chosen to minimise the loss of ringed individuals from the study population due to small-scale nest burrow movements. The recapture rates of birds do not differ significantly between the centre and edge of the Isthmus study plot, indicating that local breeding dispersal is infrequent [22]. Although larger-scale movements cannot be discounted, which might bias survival estimates, no inter-colony movements of breeding adults to or from Skomer and its neighbouring islands have been detected. The Isthmus study plot is a tiny fraction of the wider island population [21], therefore very few birds ringed as chicks are recaptured as local recruits to the study plot.

Breeding Manx shearwaters are ringed in the Isthmus study plot on Skomer Island under licence using hard-metal British Trust for Ornithology (BTO) rings. Ringing and re-sighting events are derived from two activities. Firstly, in mid to late April, adult birds on the surface of the colony are captured at night by hand to recapture previously ringed birds. Secondly, burrows in the study area are checked approximately weekly during the early incubation stage, i.e., from late May to early June, until the identity of both breeding birds and the presence of an egg is recorded. The individual sex of birds is unknown and therefore not included in our study. Empty burrows in the study area continue to be checked throughout incubation for late breeders, or the presence of non-breeding birds. Checks for hatched chicks are conducted weekly in all occupied burrows from late June to early July and continue until a chick is found to be present or the egg is deemed unviable (i.e., an egg found cold or of very low weight, and with no incubating adult on successive burrow checks during the incubation period). Each monitored breeding event is recorded to have one of three outcomes: 1) successful breeding (SB), defined by the presence of a large, well feathered chick in the study burrow in mid-August that is highly likely to fledge successfully within the next 4–6 weeks as late chick mortality is minimal [20]; 2) failed breeding (FB), defined as an egg laid (breeding attempt) that does not result in a successfully-fledged chick; and 3) non-breeding (NB), when a burrow is occupied by a pair of shearwaters but no breeding attempt is made (i.e., no egg is laid). Adult birds enter the study population in any one of these breeding states (SB, FB or NB) at an unknown age.

#### Environmental covariates

We used key foraging areas identified for Manx Shearwaters during the breeding and non-breeding season (Fig 1; [17, 18]) to select environmental covariates representing local- and large-scale climatic variation experienced over the annual cycle. Climate variables and time-lags were selected in conjunction with demographic studies on other seabirds and based on *a priori* expectations of their potential impact on the foraging behaviour and population ecology of Manx shearwaters (Table 1). To obtain climate variables at a relevant temporal scale, we calculated mean values for periods when shearwaters are known to be present in breeding or over-wintering areas (Table 1). To estimate these values at a relevant spatial scale, we extracted data using one-degree squares of latitude and longitude from the centre of the breeding and non-breeding foraging areas (Fig 1). Here, the proposed mechanism is that conditions during the summer breeding season directly influence breeding propensity and success in a given year, as well as indirectly influencing survival during the subsequent winter through carry-over effects. For the variables describing the non-breeding season, the proposed mechanism is that conditions directly influence overwinter survival in a given year, and indirectly influence the subsequent breeding propensity and success through carry-over effects.

**Table 1:** Climatic variables considered for association with Manx shearwater demography.

Location	Variable	Time period	Interpretation
**North Atlantic**	N.SST	February-March	Indirect effects on prey availability during the breeding season via zooplankton density, additionally lagged to account for delayed effects on larger prey (1-2yrs)
	N.SST_lag1_		
	N.SST_lag2_		
	N.Wind	May-August	Direct flight and foraging costs during the breeding season associated with high winds
	Summer NAO	May-August	Direct effects during the breeding season associated with adverse weather/poor conditions
	Winter NAO	December-March	Indirect effects during the breeding season via prey recruitment (see N.SST)
	Winter NAO_lag1_	December-March	
	Winter NAO_lag2_	December-March	
**South Atlantic**	S.SST	November-February	Indirect effects on prey availability during the non-breeding season via zooplankton density, additionally lagged to account for delayed effects on larger prey (1-2yrs)
	S.SST_lag1_		
	S.SST_lag2_		
	S.Wind		Direct flight and foraging costs during the non-breeding season associated with high winds
	SOI		Direct effects during the non-breeding season due to adverse weather/poor conditions

Sea surface temperatures (SST) at various time lags have been associated with food supply, adult survival and breeding success in multiple seabird populations [23–28]. We included SST in the breeding and non-breeding areas for the current year to reflect proximate effects on plankton productivity. We also included SST with one and two-year lags to reflect the time taken for environmental effects to cascade through trophic levels to seabird prey species—field observation of regurgitated stomach contents indicates that the diet of breeding Manx shearwaters predominantly consists of 8-15cm juvenile clupeid fish that are likely to be 1 to 2 years old (M. Brooke, B. Dean, H. Kirk, O. Padget, T. Guilford & C. Perrins pers. comms.). Seasonal mean wind speed was calculated as the hypotenuse of mean northerly and mean easterly wind speeds and used to reflect wind patterns in the breeding and non-breeding areas. SST and wind data were calculated from monthly averages available from NOAA’s NCEP-NCAR CDAS-1 Reanalysis data set http://iridl.ldeo.columbia.edu/SOURCES/.NOAA/.NCEP-NCAR/.CDAS-1/.MONTHLY/. Finally, to capture climatic variation at large spatial scales, we included two climatic indices identified as influential to seabird demography in the UK and South Atlantic [15, 29, 30]; the North Atlantic Oscillation (NAO) [31] and the Southern Oscillation Index (SOI) [32], respectively. North Atlantic Oscillation (NAO) measures the magnitude of the difference between low pressure systems over Iceland and high pressure over the Azores, with higher values associated with stronger westerly air flows, cooler wetter summers and milder wetter winters with more frequent and stronger storms in the North Atlantic [31]. By contrast, negative values of SOI are associated with El Niño phases of the oscillation, increased precipitation and more frequent and stronger storms in the South-West Atlantic [33]. Values of NOA and SOI were extracted from the NOAA Climate Prediction Center as monthly averaged data http://www.esrl.noaa.gov/psd/data/climateindices/list/.

### Multi-state capture–recapture models

We constructed multi-state capture-recapture models [34] using program E-SURGE v2.2.3 [35]. We used these models to jointly estimate annual apparent survival, breeding propensity and breeding success, as well as perform model selection and evaluate the influence of environmental covariates on each demographic process.

**Fig 2:**
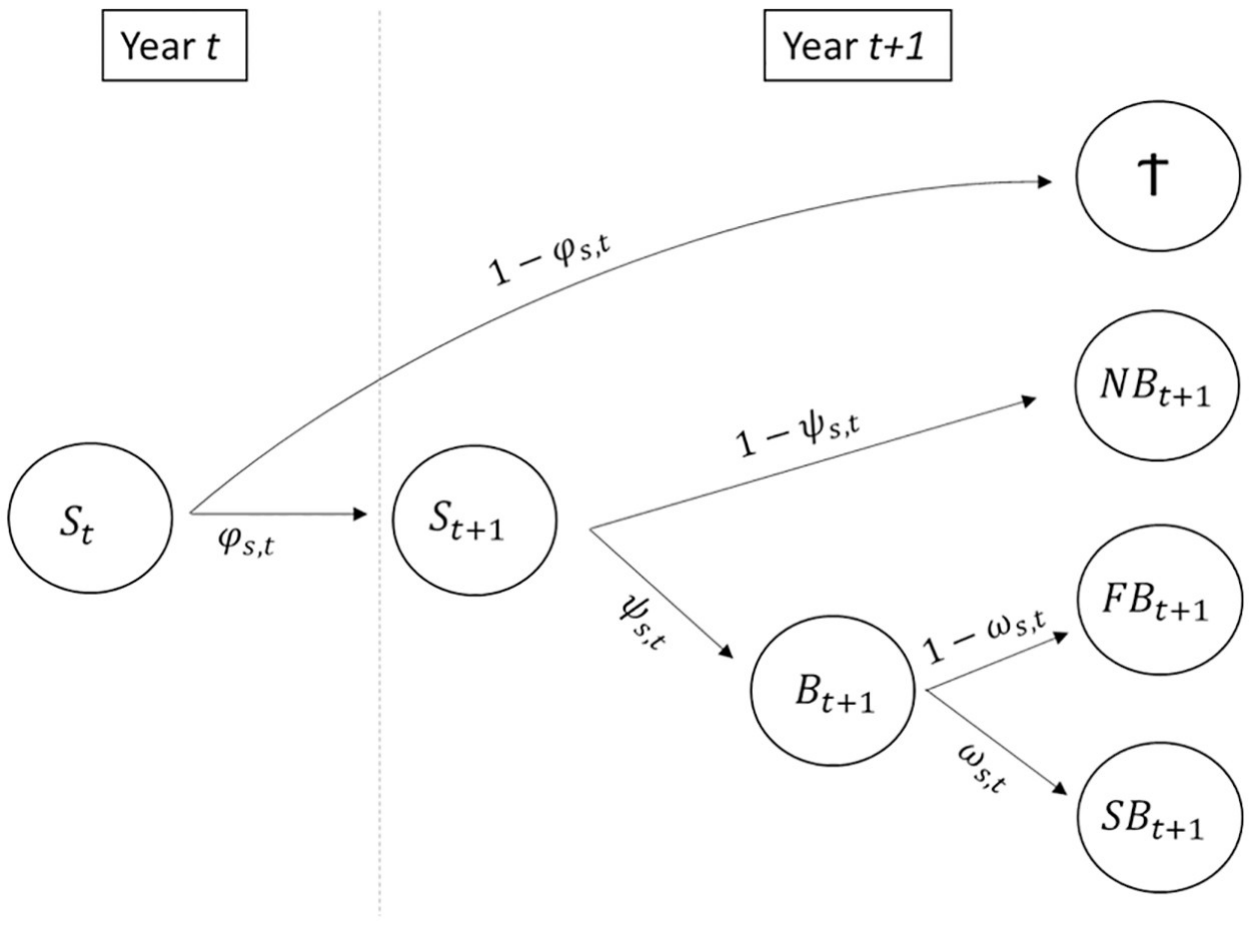
Schematic of the multi-state model structure used to estimate survival (*ϕ*), breeding propensity (*ψ*) and breeding success (*ω*) of Manx shearwaters between 1993 and 2019 on Skomer Island. At the first occasion, birds can be observed in one of three breeding states (*r*) in the model: non breeder (NB), failed breeder (FB) or successful breeder (SB). The model then describes the transition between states from year *t* to *t + 1* as three conditional steps. The first step estimates the probability of an individual in a given state (*r* = NB, FB or SB) surviving from year *t* to *t + 1* ($\phi {\;}_{t}^{r}$). The second step estimates the probability of surviving birds attempting to breed (*B*) in year *t + 1*, given their breeding state in year *t* ($\psi_{t}^{r}$). Finally, the third step estimates the probability that birds attempting to breed in year *t + 1* successfully fledge a chick, given their breeding state in year *t* ($\omega {\;}_{t}^{r}$).

The intensive monitoring of breeding attempts for Manx shearwaters at Skomer Island means that there is no uncertainty in the individual breeding states assigned annually to birds: non-breeders (NB), failed breeders (FB), and successful breeders (SB). To estimate the probability of transitioning from one state to another between years, we incorporated three conditional steps into the multi-state capture-recapture model (Fig 2). In the first step, we estimated the probability of an individual in a given state (*r* = NB, FB or SB) surviving from year *t* to *t + 1* ($\phi {\;}_{t}^{r}$). In the second step, we estimated the probability of a surviving bird attempting to breed in year *t + 1*, given its state during the previous year ($\psi_{t}^{r}$). Finally, in the third step we estimated the probability of a breeding bird in year *t + 1* successfully raising a chick to fledging, given its state during the previous year ($\omega {\;}_{t}^{r}$).

The starting multi-state model was constructed to include interactions between state and time for survival, breeding propensity and breeding success, as well as additive effects of state and time for detection probabilities. We performed multistate goodness-of-fit tests (GOF) on this model using UCARE v3.3.0 software, including transience (i.e. a lower probability of subsequent recapture of birds marked for the first time, compared to others; [36]) and trap dependence (i.e. where recapture probability is dependent on whether an individual was captured at the previous time step; [37, 38]). The GOF test revealed the presence of trap-dependence in the dataset (trap-happiness; Test M.ITEC: χ^2^ = 186.728 df = 45, P<0.001), most likely reflecting the behaviour of field observers returning to previously occupied burrows. We accounted for this lack-of-fit by employing a model structure that explicitly accounts for trap-dependence in recapture rates [38]. Remaining lack-of-fit was accounted for by using a variance inflation factor for conservative model selection, calculated as the ratio of the sum of χ2-statistics to the sum of degrees of freedom for the remaining components of the GOF test: ĉ = 499/444 = 1.12 [37].

To simplify the starting multi-state model, we used a sequential backward model selection procedure where all candidate models were nested within the starting model. In the first step, we identified the most parsimonious structure for describing variation in recapture rates. Then, with this structure included, we identified the most parsimonious structure for describing breeding propensity, breeding success, and finally, survival. The full model selection procedure is shown in S1 Table. For all model selection comparisons, the most parsimonious model was selected using the second-order Akaike Information Criterion adjusted for overdispersion (QAICc) [35, 39]. Models with a difference of less than two QAICc units were considered equivalent in their ability to describe the data [40].

In the final step of the analysis, we first identified the most influential climate covariate to each demographic process. Other covariates were then fitted alongside this and retained if they significantly improved the deviance explained by the model. To avoid collinearity amongst climate covariates, the second covariates tested were only those with low collinearity to the retained first covariate. We used the results of the backward model selection to determine if covariates should be considered as additive or interactive between state. We measured the statistical support for each covariate using ANODEV (i.e., the proportion of deviance accounted for by the inclusion of each covariate, R^2^_DEV), as [DEV(Model.)—DEV(Model_cov_)/DEV(Model.)—DEV(Model_t_)] where DEV was the deviance for models with constant (.), covariate (cov) and total temporal (t) variation [41]. When measuring the support for including a second covariate, the model including the covariate selected during the previous step was used as the constant model. A covariate explaining more than 20% of the remaining deviance was taken to be significantly influential [42]. All covariates were centred and standardised before inclusion in models. To ensure convergence of models on the global minima, models were run using repeated random initial values (‘multiple random’ option with N = 8; 55).

## Results

The dataset contained 1146 birds ringed as adults with 5028 captures between 1993 and 2019. Observations of non-breeding birds represented only 5.7% of all captures. By contrast, capture events were more balanced between the remaining two breeding states: 51.9% for successful breeders and 42.4% for failed breeding attempts. In the manuscript, we focus on reporting environmental covariates identified as significantly influential to survival, breeding propensity and breeding success (i.e., R^2^_DEV ≥ 0.20), however several other covariates achieved an R^2^_DEV between 0.10 and 0.20 indicating potential influence. Full results are presented in S2 Table.

### Survival

**Fig 3:**
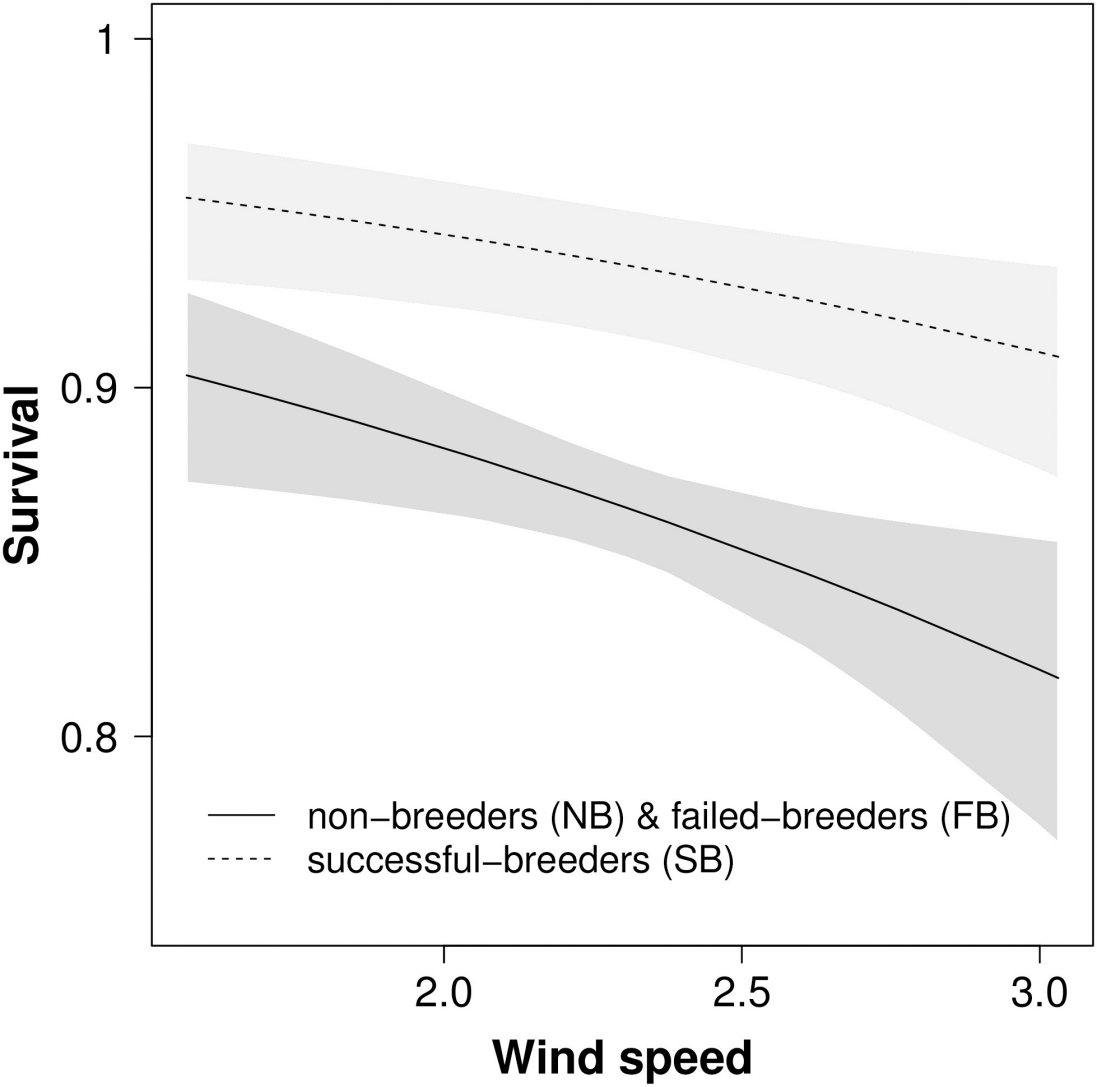
Apparent survival rates of adult Manx shearwaters decreased with increasing average wind speeds in the breeding foraging areas. Successful breeding (black line, dark grey shading shows 95% CI); grouped failed breeding and non-breeding birds (dotted line light grey shading shows 95% CI).

Model selection identified that state-specific survival rates of Manx shearwaters demonstrated very low temporal variation over the course of the study. For example, the model with constant state-specific survival, *ϕ*(NBFB + SB), was better supported than the model with time-varying survival, *ϕ*(NBFB + SB + t), (ΔQAICc = 2.10). Here, the mean values of survival differed between states, whereby successful breeders had a survival rate approximately 10% higher than failed breeders and non-breeders (*ϕ*^*SB*^ = 0.94, 95% CI 0.92–0.95; *ϕ*^*NB*&*FB*^ = 0.86, 95% CI 0.85–0.88, S1 Table). Furthermore, although temporal variation in rates of apparent survival was low (SD_NB&FB_ = 0.06, and SD_SB_ = 0.03), the step-up procedure identified that average wind force during the breeding season explained 23% of this variation (Table 2). Here, higher winds were associated with lower survival rates in successful breeders, as well as failed and non-breeders (Fig 3).

**Table 2:** Step-up ANODEV selection procedure to identify climate covariates that significantly improve the amount of deviance explained by the multi-state capture-recapture model describing rates of survival (*ϕ*), breeding propensity (*ψ*) and breeding success (*ω*) of Manx shearwaters breeding on Skomer Island 1993–2019.

Parameter	Models	K	Dev	QAICc	ΔQAICc	R^2^_DEV
*ϕ*	NBFB + SB	138	17182.65	15625.49	0.00	
	NBFB + SB + t	163	17145.80	15645.74	20.25	
	NBFB + SB + t*N_wind	139	17174.25	15620.11	-5.38	0.23
*ψ* ^*SB*^	NB*t + FB*t + SB	138	17189.60	15631.70	0.00	
	NB*t + FB*t + SB*t	163	17145.80	15645.74	14.04	
	NB*t + FB*t + SB*t*wNAO_lag2_	139	17173.38	15619.33	-12.37	0.37

*R*^2^_DEV gives the estimated explanatory power of the climate variable to explain temporal variation in the specified rate. Only significant variables are shown, i.e., R^2^_DEV ≥ 0.20, (42). We used the results of the backward model selection to describe recapture probabilities and determine if covariates should be considered as additive or interactive between state (S1 Table). ΔQAIC_c_ gives the difference in QAIC_c_ between the model with the covariate effect and a model in which the demographic rate involved was state-dependent but time-invariant (i.e. the first line model of each box). See Table 1 for description of climate variables (N_wind and wNAO_lag2_). Time (t) is either additive (+) or interactive (*), and birds are considered in one of three states each year: non breeder (NB), failed breeder (FB) or successful breeder (SB).

### Breeding propensity

**Fig 4:**
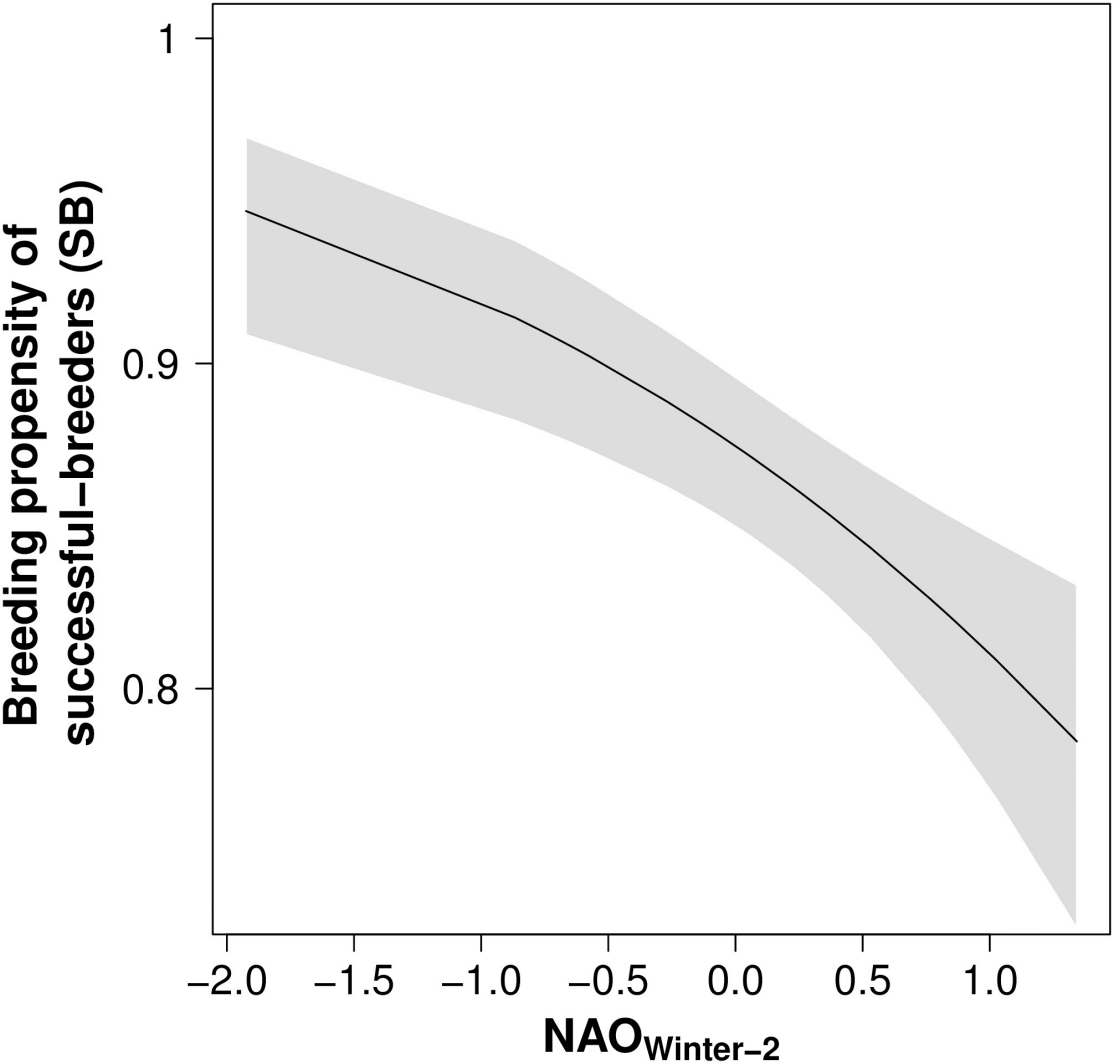
Breeding propensity of successfully breeding Manx shearwaters decreased with increasing values of the North Atlantic Oscillation (NAO) during the non-breeding season (from December to March) with a two-year lag. Grey shading shows 95% CI.

Breeding propensity was strongly related to breeding state in the previous year. The mean probability of non-breeders resuming breeding was low (*ψ*^*NB*^ = 0.21, 95% CI 0.18–0.24). By contrast, the mean probability of failed and successful breeders attempting to breed in successive years was considerably higher (*ψ*^*SB*^ = 0.87, 95% CI 0.84–0.89; *ψ*^*FB*^ = 0.70, 95% CI 0.67–0.73). Significant annual variation in breeding propensity was only identified in successful breeding and non-breeding birds (S1 Table, SD_SB_ = 0.16; SD_NB_ = 0.14; SD_FB_ = 0.12). The step-up procedure identified that breeding propensity in successful breeders decreased at higher values of winter North Atlantic Oscillation with a 2-year lag, whereby 37% of temporal variation in the breeding propensity of successful breeders was explained by this variable (Fig 4, Table 2). However, none of the tested environmental covariates had a significant effect on breeding propensity in non-breeding and failed-breeding birds. From the climatic variables considered, wind speed in the breeding area explained the highest amount of deviance for failed breeders (R^2^_DEV = 0.14, S2 Table), and wind speed in the wintering area explained the highest amount of deviance for non-breeding birds (R^2^_DEV = 0.18, S2 Table).

### Breeding success

Mean estimates of breeding success in year *t* were equivalent for birds with failed or non-breeding status in year *t—1* (*ω*_*FB*_ = *ω*_*NB*_ = 0.56, 95% CI 0.53–0.59). By contrast, breeding success was 12.5% higher for birds that were successful breeders in year *t– 1* (*ω*_*SB*_ = 0.64, 95% CI 0.62–0.67). Model selection indicated that temporal variation in breeding success was best described as additive with time across states. In other words, individuals in the three states experienced synchronous annual breeding success (S1 Table). However, despite strong temporal variation in breeding success (SD = 0.16), none of the tested environmental variables were identified as significantly influential. From the climatic variables considered, winter NAO values the previous year explained the highest amount of deviance (R^2^_DEV = 0.09, S2 Table).

### Detection

Recapture rates were additive between states and considerably higher for trap-aware breeding individuals (*p*^*SB*^ = 0.97, 95% CI 0.92–0.99; *p*^*FB*^ = 0.94, 95% CI 0.86–0.98), compared to trap-aware non-breeding individuals (*p*^*NB*^ = 0.11, 95% CI 0.09–0.14; S1 Table). Annual variation in recapture rates was low ($p_{a}^{SB}\text{SD}=0.03$, $p_{a}^{NB}\text{SD}=0.05$,$p_{a}^{FB}\text{SD}=0.02$; $p_{a}^{SB}\text{SD}=0.06$; $p_{a}^{NB}\text{SD}=0.02$, $p_{a}^{FB}\text{SD}=0.05$; whereby subscript *a* and *u* refers to trap-aware and trap-unaware birds, respectively).

## Discussion

In this study, we examine whether individual differences in breeding state may alter spatio-temporal exposure to weather conditions and generate heterogeneity in the demography of a trans-Atlantic migrant seabird, the Manx shearwater. We find that demographic profiles vary between individuals, whereby more successful reproduction is associated with higher rates of survival and breeding propensity. We also show that environmental effects can differentially influence individuals in different breeding states (i.e., non-breeder, failed breeder or successful breeder), such that environmental fluctuations and climate change could alter the breeding frequency of individuals with different demographic profiles, thereby driving a complex and less predictable population response.

### 1. Demographic profiles

We identified heterogeneity in the demographic profiles of Manx shearwaters breeding on Skomer Island. Birds that successfully raised a chick in the previous year were 10% more likely to survive to the subsequent year, at least 20% more likely to breed in that year and 12.5% more likely to successfully raise another chick to fledging. For the successfully breeding birds, this demographic pattern indicates that at the population level Manx shearwaters generally do not experience fitness costs associated with reproduction. This pattern is reported in other seabirds and long-lived species [43, 44], although is not ubiquitous [45]. We also found that non-breeders had a higher probability of remaining as non-breeders in subsequent years, compared to breeders. This demographic profile has been previously considered to represent individuals of lower intrinsic “quality” [46]. However, an alternative explanation is that populations are heterogenous and comprised of individuals that exist along a continuum of quantitative traits [47, 48].

A previous capture-recapture study on Manx shearwaters breeding in a separate subplot on Skomer Island reports that non-breeding birds were less likely to fail or skip breeding in the following year, compared to successful breeders [49]. This suggests that non-breeding Manx shearwaters are employing an adaptive ‘gap year’ to recover and increase future breeding potential, as opposed to being in a ‘non-breeding trap’ as indicated by our study. In contrast to our study, birds selected for the previous analysis all entered the study as successful breeders [49], thus potentially creating an unrepresentative sample lacking the regular non-breeders we find in our study population. Differences in study plot locations may also explain distinctions in the resulting demographic rates. The Isthmus study plot used in our study is fringed on two sides by coastal cliff-edge containing many burrows of Atlantic Puffins *Fratercula arctica*, with potential for interspecies competition for nesting burrows [50]. By contrast, the study plot used in the previous study has a lower density of nesting puffins [49]. Studies of competition between shearwaters and puffins on Skomer Island find equal rates of burrow displacement between these species, so it remains unclear whether direct competition with puffins may be influencing the different strategies reported. A repeat of Ashcroft’s [51] work might be insightful here, as the puffin population has increased markedly since the 1970s [52]. No correlation between breeding success and distance to cliff edge has been detected in the Isthmus study population [22], however, non-breeding shearwaters may find it difficult to defend a nest burrow and choose to disperse out of the study area, potentially biasing our estimation of rates of survival for non-breeders as well as their transition rates from non-breeder back to breeder. However, the estimated rates of survival and breeding success for birds identified as non-breeders in the previous year were identical to birds identified as being failed breeders suggesting either that biases were minimal, or failed breeders were also dispersing. Increased prospecting following large-scale breeding failure has been previously reported in cliff-nesting seabirds [53], however, similar social cues are likely to be less prevalent in burrow nesting species, such as Manx shearwaters.

### 2. Effects of environmental covariates

We found that higher wind speeds during the breeding season decreased rates of survival during the subsequent winter. Strong wind speeds might seem ideal for birds reliant on dynamic soaring flight such as Procellariiformes [54, 55]. For example, wind is known to influence seabird flying [56–58] and transitions between different behaviours [59]. Flapping flight employed in low wind speeds is also associated with increased metabolic flying costs, compared to flying in stronger wind conditions [60]. However, flying into a headwind has a greater energetic cost compared to flying with a tailwind [61]. Severe weather events have also been linked to reduced rates of provisioning and breeding success, possibly due to difficulties foraging and locating prey [62–66]. Therefore, the identified negative relationship between seabird survival and wind speed during the breeding season may reflect the energetic costs associated with foraging in adverse conditions under central place constraint. This finding also implies that individual Manx shearwaters experience a survival cost associated with reproduction (or central place constraint for non-breeders) during years with severe wind conditions. The detection of reproductive costs only during severe environmental conditions is reported in terrestrial mammals and birds [67–71], although studies showing this in seabirds are limited.

That the same relationship between wind speed and survival was identified for birds in all breeding states suggests that birds maintain similar foraging routines during the breeding season. In agreement, the foraging distribution of black-browed albatross *Thalassarche melanophris* has been shown not to significantly differ between failed and successful breeding birds [72]. Tracking Manx shearwaters in all breeding states during the breeding season and evaluating whether activity budgets and time spent transiting to and from the colony differs between breeding states may elucidate the mechanisms linking wind speed during the breeding season to survival during the subsequent winter.

The relationship between winter NAO with a two-year lag and breeding propensity was only identified for birds that were successful breeders in the previous year. That environmental variables differentially influence the breeding propensity of birds in different breeding states (i.e., non-breeder, failed breeder or successful breeder) suggests some degree of spatio-temporal variation in habitat use between these groups at particular points in the annual cycle. Earlier time of arrival is linked to increased rates of breeding success in Australasian gannets *Morus serrator* [73], and previous studies of Manx shearwaters breeding on Skomer Island report that an earlier breeding phenology increases breeding success [49]. Consequently, it may be that successful breeders arrive earlier at the colony to initiate breeding and experience different environmental conditions during this time. Alternatively, successful breeders may adopt different foraging strategies prior to breeding, foraging in areas that are segregated from failed and non-breeders. Further evaluation of tracking data collected from Manx shearwaters breeding on Skomer island may clarify this.

In this study, we examine the potential effects of local-and large-scale environmental variation on the demography of Manx shearwaters breeding on Skomer Island and evaluate whether relationships differentially influence individuals in different breeding states. We find that Manx shearwaters exhibit heterogenous demographic profiles, and that reproduction has an associated survival cost in years where severe wind conditions occur during the breeding season. We also show that environmental drivers of breeding propensity differ for birds that were successful breeders in the previous year, suggesting spatio-temporal variation in habitat use occurs at key points in the annual cycle. Climate change therefore not only has the potential to increase costs of reproduction and alter population-level rates of survival, but also alter the breeding frequency of individuals with different demographic profiles, thereby driving a complex and less predictable population response.

## Supporting information

S1 Fig Modelling survival and reproduction parameters for Manx shearwaters captured between 1993 and 2019 on Skomer Island, Wales, UK: Elementary matrices of state–state transitions and events. (DOCX)

S2 Fig Estimates of annual variation in demographic rates over the study period (1993–2019). (DOCX)

S1 Table The backward model selection procedure for identifying the most parsimonious structure of the multi-state model for describing recapture (*p*), breeding probability (*ψ*), breeding success (*ω*) and survival (*ϕ*) probabilities of non-breeding (NB), failed breeding (FB) or successful breeding (SB) Manx shearwaters (1993–2019). (DOCX)

S2 Table Step-up model selection procedure, ANODEV, to identify environmental covariates that significantly improve the amount of deviance explained by the model for apparent survival (*ϕ*), breeding probability (*ψ*) and breeding success (*ω*) of Manx shearwaters breeding on Skomer Island (1993–2019). (DOCX)
