# Supplementary material for: Demographic profiles and environmental drivers of variation relate to individual breeding state in a long-lived trans-oceanic migratory seabird, the Manx shearwater
Source: PLoS One. 2021 Dec 16;16(12):e0260812. doi: 10.1371/journal.pone.0260812 (PMC8675709; doi:10.1371/journal.pone.0260812)
Supplement: S1 Fig — (DOCX) [file pone.0260812.s001.docx]

**S1 Fig. Modelling survival and reproduction parameters for Manx shearwaters captured between 1993 and 2019 on Skomer Island, Wales, UK: elementary matrices of state–state transitions and events.**

The rows correspond to the departure state at time t and the columns to the state of arrival at time t+1. From the initial state of departure, the individual's state was successively updated through decomposed modelling steps: (1) survival, (2) breeding propensity and (3) breeding success. The observation process was modelled with two steps: (1) detection taking trap-dependence into account and (2) observation. Notation as follows: initial state probabilities ($\Pi$), recapture ($p$) of trap-aware (A) and trap-unaware (U) birds, breeding propensity ($\psi$), breeding success ($\omega$) and survival probabilities () of non-breeding (NB), failed breeding (FB) or successful breeding (SB) birds. Finally, ($ϯ$) corresponds to the last state “dead”.

**Initial states of departure:**

$$\begin{matrix} {A NB}_{t} & {U NB}_{t} & {A FB}_{t} & {U FB}_{t} & {A SB}_{t} & {U SB}_{t} & ϯ_{t} \\ & & & & & & \\ \Pi_{NB} & 0 & \Pi_{FB} & 0 & \Pi_{SB} & 0 & 0 \end{matrix}$$

**Step 1: Survival** $\boldsymbol{\varphi}$

$\begin{matrix} & {A NB}_{t+1}- & {U NB}_{t+1}- & {A FB}_{t+1}- & {U FB}_{t+1}- & {A SB}_{t+1}- & {U SB}_{t+1}- & ϯ_{t+1}- \\ & & & & & & & \\ {A NB}_{t} & \varphi_{NB} & 0 & 0 & 0 & 0 & 0 & 1-\varphi_{NB} \\ {U NB}_{t} & 0 & \varphi_{NB} & 0 & 0 & 0 & 0 & 1-\varphi_{NB} \\ {A FB}_{t} & 0 & 0 & \varphi_{FB} & 0 & 0 & 0 & {1-\varphi}_{FB} \\ {U FB}_{t} & 0 & 0 & 0 & \varphi_{FB} & 0 & 0 & {1-\varphi}_{FB} \\ {A SB}_{t} & 0 & 0 & 0 & 0 & \varphi_{SB} & 0 & {1-\varphi}_{SB} \\ {U SB}_{t} & 0 & 0 & 0 & 0 & 0 & \varphi_{SB} & {1-\varphi}_{SB} \\ ϯ_{t} & 0 & 0 & 0 & 0 & 0 & 0 & 1 \end{matrix}$

**Step 2: Breeding probability** $\boldsymbol{\psi}$

$$\begin{matrix} & {A toNB}_{t+1}- & {U toNB}_{t+1}- & {A FBtoB}_{t+1}- & {U FBtoB}_{t+1}- & {A SBtoB}_{t+1}- & {U SBtoB}_{t+1}- & {A NBtoB}_{t+1}- & {U NBtoB}_{t+1}- & ϯ_{t+1}- \\ & & & & & & & & & \\ {A NB}_{t+1}- & {1-\psi}_{NB} & 0 & 0 & 0 & 0 & 0 & \psi_{NB} & 0 & 0 \\ {U NB}_{t+1}- & 0 & {1-\psi}_{NB} & 0 & 0 & 0 & 0 & 0 & 0 & 0 \\ {A FB}_{t+1}- & {1-\psi}_{FB} & 0 & \psi_{FB} & 0 & 0 & 0 & 0 & \psi_{NB} & 0 \\ {U FB}_{t+1}- & 0 & {1-\psi}_{FB} & 0 & \psi_{FB} & 0 & 0 & 0 & 0 & 0 \\ {A SB}_{t+1}- & {1-\psi}_{SB} & 0 & 0 & 0 & \psi_{SB} & 0 & 0 & 0 & 0 \\ {U SB}_{t+1}- & 0 & {1-\psi}_{SB} & 0 & 0 & 0 & \psi_{SB} & 0 & 0 & 0 \\ ϯ_{t+1}- & 0 & 0 & 0 & 0 & 0 & 0 & 0 & 0 & 1 \end{matrix}$$

**Step 3: Breeding success** $\boldsymbol{\omega}$

$\begin{matrix} & {A NB}_{t+1}- & {U NB}_{t+1}- & {A FB}_{t+1}- & {U FB}_{t+1}- & {A SB}_{t+1}- & {U SB}_{t+1}- & ϯ_{t+1}- \\ & & & & & & & \\ {A toNB}_{t+1}- & 1 & 0 & 0 & 0 & 0 & 0 & 0 \\ {U toNB}_{t+1}- & 0 & 1 & 0 & 0 & 0 & 0 & 0 \\ {A FBtoB}_{t+1}- & 0 & 0 & {1-\omega}_{FB} & 0 & \omega_{FB} & 0 & 0 \\ {U FBtoB}_{t+1}- & 0 & 0 & 0 & {1-\omega}_{FB} & 0 & \omega_{FB} & 0 \\ {A SBtoB}_{t+1}- & 0 & 0 & {1-\omega}_{SB} & 0 & \omega_{SB} & 0 & 0 \\ {U SBtoB}_{t+1}- & 0 & 0 & 0 & {1-\omega}_{SB} & 0 & \omega_{SB} & 0 \\ {A NBtoB}_{t+1}- & 0 & 0 & {1-\omega}_{NB} & 0 & \omega_{NB} & 0 & 0 \\ {U NBtoB}_{t+1}- & 0 & 0 & 0 & {1-\omega}_{NB} & 0 & \omega_{NB} & 0 \\ ϯ_{t+1}- & 0 & 0 & 0 & 0 & 0 & 0 & 1 \end{matrix}$

**Step 4: Recapture with trap-dependence** $\boldsymbol{p}$

$$\begin{matrix} & {A NB}_{t+1}+ & {U NB}_{t+1}+ & {A FB}_{t+1}+ & {U FB}_{t+1}+ & {A SB}_{t+1}+ & {U SB}_{t+1}+ & ϯ_{t+1}+ \\ & & & & & & & \\ {A NB}_{t+1}- & p_{ANB} & 1-p_{ANB} & 0 & 0 & 0 & 0 & 0 \\ {U NB}_{t+1}- & p_{UNB} & 1-p_{UNB} & 0 & 0 & 0 & 0 & 0 \\ {A FB}_{t+1}- & 0 & 0 & p_{AFB} & 1-p_{AFB} & 0 & 0 & 0 \\ {U FB}_{t+1}- & 0 & 0 & p_{UFB} & 1-p_{UFB} & 0 & 0 & 0 \\ {A SB}_{t+1}- & 0 & 0 & 0 & 0 & p_{ASB} & 1-p_{ASB} & 0 \\ {U SB}_{t+1}- & 0 & 0 & 0 & 0 & p_{USB} & 1-p_{USB} & 0 \\ ϯ_{t+1}- & 0 & 0 & 0 & 0 & 0 & 0 & 1 \end{matrix}$$

**Observation**

$$\begin{matrix} & '0' & '1' & '2' & '3' \\ & & & & \\ {A NB}_{t} & 0 & 1 & 0 & 0 \\ {U NB}_{t} & 1 & 0 & 0 & 0 \\ {A FB}_{t} & 0 & 0 & 1 & 0 \\ {U FB}_{t} & 1 & 0 & 0 & 0 \\ {A SB}_{t} & 0 & 0 & 0 & 1 \\ {U SB}_{t} & 1 & 0 & 0 & 0 \\ ϯ_{t} & 1 & 0 & 0 & 0 \end{matrix}$$
