# Supplementary material for: Demographic profiles and environmental drivers of variation relate to individual breeding state in a long-lived trans-oceanic migratory seabird, the Manx shearwater
Source: PLoS One. 2021 Dec 16;16(12):e0260812. doi: 10.1371/journal.pone.0260812 (PMC8675709; doi:10.1371/journal.pone.0260812)
Supplement: S2 Fig — (DOCX) [file pone.0260812.s002.docx]

**S2 Fig.** **Estimates of annual variation in demographic rates over the study period (1993-2019).**

A) apparent survival rates of non-breeding and failed breeding Manx shearwaters, B) breeding success probabilities of non-breeding and failed breeding Manx shearwaters, C) breeding propensity of non-breeding birds and successful breeding birds the previous year and D) detection probabilities of aware failed breeding birds. For each year we represented the mean value (symbol) and the 95% CI. To simplify reading, we only represented probabilities for one state when time variation was modelled as an additive effect over the different states; i.e. A), C) and D).
