# Supplementary material for: Demographic profiles and environmental drivers of variation relate to individual breeding state in a long-lived trans-oceanic migratory seabird, the Manx shearwater
Source: PLoS One. 2021 Dec 16;16(12):e0260812. doi: 10.1371/journal.pone.0260812 (PMC8675709; doi:10.1371/journal.pone.0260812)
Supplement: S1 Table — (DOCX) [file pone.0260812.s003.docx]

**S1 Table. The backward model selection procedure for identifying the most parsimonious structure of the multi-state model for describing recapture ($p$), breeding probability ($\psi$), breeding success ($\omega$) and survival () probabilities of non-breeding (NB), failed breeding (FB) or successful breeding (SB) Manx shearwaters (1993-2019).**

Starting with the fully parametrized model, we tested for effects of time (t) and states (r). Effects were either additive (+) or included as an interaction with another parameter (*). Finally, when the parameter did not depend of the state nor varied in time it was noted (.). All models also incorporated an additive trap-dependence effect in recapture probabilities (td). K = number of parameters; Dev = model deviance; *w* = model weight. Within each parameter, models are ordered by QAIC_C_. Model fit is assessed using the lowest QAIC_C_ with the difference between the best candidate model and other models specified (ΔQAICc). Best fitting model shown in bold.

|  | **Detection** | **Breeding probability** | **Breeding success** | **Survival** | **K** | **Dev** | **QAICc** | **ΔQAICc** | ***w*** |
| --- | --- | --- | --- | --- | --- | --- | --- | --- | --- |
| **1) Recapture** | **r + t + td** | r | r | r*t | **115** | **17637.44** | **15983.15** | **0** | **1** |
|  | NB + FBSB + t + td |  |  |  | 114 | 17699.43 | 16036.4 | 53.25 | 0 |
|  | r + td |  |  |  | 90 | 17759.96 | 16040.42 | 57.27 | 0 |
|  | NBFB + SB + t + td |  |  |  | 114 | 17830.94 | 16153.82 | 170.67 | 0 |
|  | t + td |  |  |  | 112 | 17855.28 | 16171.37 | 188.22 | 0 |
|  | NBSB + FB + t + td |  |  |  | 114 | 17850.80 | 16171.55 | 188.40 | 0 |
|  | td |  |  |  | 88 | 17922.65 | 16181.54 | 198.39 | 0 |
| **2) Breeding probability** | r + t + td | **NB*t + FB + SB*t** | r | r*t | **165** | **17499.19** | **15965.54** | **0** | **0.99** |
|  |  | r*t |  |  | 190 | 17449.02 | 15974.49 | 8.95 | 0.01 |
|  |  | r |  |  | 115 | 17637.44 | 15983.14 | 17.6 | 0 |
|  |  | NB*t + FB*t + SB |  |  | 165 | 17519.00 | 15983.23 | 17.69 | 0 |
|  |  | NB + FB*t + SB*t |  |  | 165 | 17527.24 | 15990.59 | 25.05 | 0 |
|  |  | r + t |  |  | 140 | 17612.68 | 16013.68 | 48.14 | 0 |
|  |  | NB*t + FBSB*t |  |  | 164 | 17579.05 | 16034.71 | 69.17 | 0 |
|  |  | t |  |  | 138 | 17726.13 | 16110.75 | 145.21 | 0 |
|  |  | . |  |  | 113 | 17800.63 | 16124.66 | 159.12 | 0 |
|  |  | NBFB*t + SB*t |  |  | 164 | 17685.53 | 16129.78 | 164.24 | 0 |
|  |  | NBSB*t + FB*t |  |  | 164 | 17687.26 | 16131.32 | 165.78 | 0 |
| **3) Breeding success** | r + t + td | NB*t + FB + SB*t | **NBFB + SB + t** | r*t | **189** | **17153.14** | **15708.14** | **0** | **0.72** |
|  |  |  | r + t |  | 190 | 17153.22 | 15710.38 | 2.24 | 0.24 |
|  |  |  | NBFB*t + SB*t |  | 214 | 17098.77 | 15713.88 | 5.74 | 0.04 |
|  |  |  | r*t |  | 240 | 17062.35 | 15738.41 | 30.27 | 0 |
|  |  |  | NBSB*t + FB*t |  | 214 | 17129.41 | 15741.23 | 33.09 | 0 |
|  |  |  | t |  | 188 | 17198.81 | 15746.77 | 38.63 | 0 |
|  |  |  | FBSB*t + NB*t |  | 214 | 17148.10 | 15757.92 | 49.78 | 0 |
|  |  |  | NBFB + SB*t |  | 189 | 17243.33 | 15788.68 | 80.54 | 0 |
|  |  |  | NBFB*t + SB |  | 189 | 17387.51 | 15917.41 | 209.27 | 0 |
|  |  |  | NBFB + SB |  | 164 | 17498.15 | 15962.48 | 254.34 | 0 |
|  |  |  | r |  | 165 | 17499.19 | 15965.54 | 257.40 | 0 |
|  |  |  | . |  | 163 | 17523.56 | 15983.03 | 274.89 | 0 |
| **4) Survival** | r + t + td | NB*t + FB + SB*t | NBFB + SB + t | **NBFB + SB** | **113** | **17252.88** | **15635.6** | **0** | **0.62** |
|  |  |  |  | NBFB + SB + t | 138 | 17196.32 | 15637.7 | 2.10 | 0.22 |
|  |  |  |  | NBFB*t + SB | 138 | 17198.23 | 15639.41 | 3.81 | 0.09 |
|  |  |  |  | . | 139 | 17196.30 | 15639.8 | 4.20 | 0.08 |
|  |  |  |  | NBFB + SB*t | 138 | 17223.78 | 15662.22 | 26.62 | 0 |
|  |  |  |  | NBFB*t + SB*t | 163 | 17169.79 | 15667.16 | 31.56 | 0 |
|  |  |  |  | NBSB*t + FB*t | 163 | 17180.97 | 15677.14 | 41.54 | 0 |
|  |  |  |  | r*t | 189 | 17153.14 | 15708.14 | 72.54 | 0 |
|  |  |  |  | r + t | 114 | 17342.94 | 15718.1 | 82.50 | 0 |
|  |  |  |  | NB*t + FBSB*t | 163 | 17309.01 | 15791.46 | 155.86 | 0 |
