# Supplementary material for: Demographic profiles and environmental drivers of variation relate to individual breeding state in a long-lived trans-oceanic migratory seabird, the Manx shearwater
Source: PLoS One. 2021 Dec 16;16(12):e0260812. doi: 10.1371/journal.pone.0260812 (PMC8675709; doi:10.1371/journal.pone.0260812)
Supplement: S2 Table — (DOCX) [file pone.0260812.s004.docx]

**S2 Table. Step-up model selection procedure, ANODEV, to identify environmental covariates that significantly improve the amount of deviance explained by the model for apparent survival (), breeding probability ($\psi$) and breeding success ($\omega$) of Manx shearwaters breeding on Skomer Island** **1993-2019.**

We used the results of the backward model selection to describe recapture probabilities and determine if covariates should be considered as additive, interactive or grouped between states: survival and breeding success differed between successful breeders (SB) and the two other breeding states (failed breeders FB and non-breeders NB) with an additive effect of time; breeding probability was interactive between state and time. ∆∆QAIC_c_ gives the difference in QAIC_c_ between the model with the covariate effect and a model in which the demographic rate involved was state-dependent but time-invariant. R^2^_DEV gives the estimated explanatory power of the covariate to explain variation in the specified rate. Finally, the third column indicates the sign of the relationship. When a covariate has a significant effect on the tested rate (R^2^_DEV ≥ 0.2), models were run again considering the model with the significant covariate as the constant model. Empty cells in the second step of covariate selection correspond to variables retained in the first step. Climate covariates were: sNAO = Summer North Atlantic Oscillation, wNAO = Winter North Atlantic Oscillation, wNAO_lag1_ = wNAO lagged 1 year, N.SST/S.SST = Northern/Southern sea surface temperature (with subscripts indicating a 1- or 2-year lag), N.wind/S.wind = Northern/Southern wind force; SOI = Southern Oscillation Index (Table 1). Significant covariates highlighted in bold (R2_DEV ≥ 0.2), covariates with an R2_DEV ≥ 0.1 shaded in light grey.

|  | **φ** | | | **φ(N_wind)** | | | **ω** | | | **ψNB** | | | **ψFB** | | | **ψSB** | | | **ψSB(wNAO-1)** | | |
| --- | --- | --- | --- | --- | --- | --- | --- | --- | --- | --- | --- | --- | --- | --- | --- | --- | --- | --- | --- | --- | --- |
| **Covariate** | ΔQAICc | R^2^_DEV | sign | ΔQAICc | R^2^_DEV | sign | ΔQAICc | R^2^_DEV | sign | ΔQAICc | R^2^_DEV | sign | ΔQAICc | R^2^_DEV | sign | ΔQAICc | R^2^_DEV | sign | ΔQAICc | R^2^_DEV | sign |
| N_SST_0 | 1.10 | 0.03 | + | 2.12 | 0.00 | + | -13.67 | 0.05 | - | 0.10 | 0.03 | + | 1.80 | 0.01 | - | 0.16 | 0.05 | + | 0.33 | 0.07 | + |
| N_SST-1 | 0.99 | 0.03 | + | 1.45 | 0.03 | + | -0.69 | 0.01 | + | -2.00 | 0.07 | - | 0.51 | 0.04 | + | 1.17 | 0.02 | - | -1.25 | 0.14 | - |
| N_SST-2 | 0.15 | 0.06 | + | 0.11 | 0.08 | + | -17.67 | 0.06 | - | 1.60 | 0.01 | - | 1.23 | 0.02 | + | -2.83 | 0.13 | - | -1.84 | 0.16 | - |
| N_wind | **-5.38** | **0.23** | **-** |  |  |  | -6.01 | 0.03 | - | 1.99 | 0.00 | - | -4.27 | 0.14 | + | 2.00 | 0.00 | - | 2.10 | 0.00 | - |
| sNAO | 0.86 | 0.04 | + | 2.16 | 0.00 | + | -19.94 | 0.07 | - | 1.84 | 0.00 | - | 2.10 | 0.00 | - | 0.54 | 0.04 | + | -1.38 | 0.14 | + |
| wNAO | -1.75 | 0.12 | + | 0.42 | 0.07 | + | -3.99 | 0.02 | + | 0.48 | 0.03 | + | 1.87 | 0.01 | - | 1.49 | 0.02 | - | 1.08 | 0.04 | - |
| wNAO-1 | 1.69 | 0.01 | + | 2.16 | 0.00 | + | 1.69 | 0.00 | + | 0.45 | 0.03 | + | 1.93 | 0.00 | - | -2.16 | 0.11 | **-** | 0.24 | 0.08 | - |
| wNAO-2 | -0.49 | 0.08 | + | 0.43 | 0.07 | + | -27.70 | 0.09 | + | -0.85 | 0.05 | + | -0.91 | 0.07 | - | **-12.37** | **0.37** | **-** |  |  |  |
| S_SST_0 | 1.93 | 0.01 | - | 0.66 | 0.06 | + | -1.17 | 0.01 | + | -4.41 | 0.11 | - | 2.18 | 0.00 | + | 1.85 | 0.01 | + | -1.92 | 0.16 | - |
| S_SST-1 | 1.94 | 0.01 | + | 2.06 | 0.00 | - | -1.47 | 0.01 | + | -7.85 | 0.16 | + | 2.18 | 0.00 | + | -1.33 | 0.09 | + | 0.05 | 0.08 | + |
| S_SST-2 | 0.47 | 0.05 | - | 0.30 | 0.07 | - | -15.92 | 0.06 | - | 1.12 | 0.02 | - | 0.17 | 0.04 | - | 1.97 | 0.00 | + | 2.05 | 0.00 | - |
| S_wind | 1.53 | 0.02 | + | 1.56 | 0.02 | + | -0.28 | 0.01 | + | -8.81 | 0.18 | - | 2.28 | 0.00 | + | 0.82 | 0.03 | + | 0.40 | 0.07 | + |
| SOI | 0.19 | 0.01 | + | 0.80 | 0.05 | + | -1.85 | 0.01 | - | 0.40 | 0.03 | - | 0.08 | 0.05 | - | 2.00 | 0.00 | - | 1.79 | 0.01 | - |
